# DAILY SLEEP QUALITY AND EXECUTIVE FUNCTIONING PERFORMANCE ACROSS TWO WEEKS IN INDIVIDUALS APPROACHING MID LIFE

**DOI:** 10.1093/geroni/igad104.0870

**Published:** 2023-12-21

**Authors:** Tina Vo, Anqing Zheng, Elizabeth Muñoz, Sally Wadsworth, Martin Sliwinski, Chandra Reynolds

**Affiliations:** University of California Riverside, Riverside, California, United States; University of Colorado Boulder, Boulder, Colorado, United States; The University of Texas at Austin, Austin, Texas, United States; University of Colorado Boulder, Boulder, Colorado, United States; Pennsylvania State University, State College, Pennsylvania, United States; University of Colorado Boulder, Boulder, Colorado, United States

## Abstract

Sleep quality (SQ)-cognition associations are prevalent across aging, with stronger associations observed in midlife. Whether these associations persist within micro-level timescales (e.g., day-to-day) is unclear, particularly in individuals approaching midlife. Burst data from a subsample of the Colorado Adoption/Twin Study of Lifespan behavioral development and cognitive aging (CATSLife1) who participated in the ambulatory smartphone substudy (N=440, Mage=35.78 range=28.07-51.32, %Female=57.7%) were utilized to examine associations between self-reported daily SQ and cognitive performance, measured up to three times a day for 14 days, while accounting for age, sex, and educational attainment. There was a small positive age effect on average SQ (e.g., d=.18 comparing 36 versus 46 year-olds). Separable between-person and within-person time varying covariate effects were extracted for SQ to capture individual differences in typical levels versus variations from one’s typical pattern. The between-person SQ was individually significant and positive (B=0.023, se=0.009, p=0.019) suggesting better Stroop accuracy for each unit increase in SQ. Daily differences from one’s average SQ did not additionally predict daily Stroop performance suggesting the salience of general quality. Estimates suggest higher accuracy for individuals who report higher average SQ (d=.19, average SQ versus +2SD above average SQ). However, there were no Age by SQ effects on Stroop accuracy. Additional SQ aspects (e.g., sleep latency and sleep maintenance) are explored. Examining sleep quality-cognition associations across micro-level timescales are salient to discern whether and how effects accumulate and contribute to performance differences over larger timescales in individuals approaching midlife.

